# Concerns of CropLife America Regarding the Application and Use of the U.S. EPA’s Toxicity Reference Database

**DOI:** 10.1289/ehp.0900951

**Published:** 2009-10

**Authors:** Erik Janus

**Affiliations:** Science & Regulatory Affairs, CropLife America, Washington, DC, E-mail:EJanus@croplifeamerica.org

In a recent article in *EHP*, Martin et al. (2009) reported classifying the relative toxicity of chemicals using the U.S. EPA’s (U.S. Environmental Protection Agency) Toxicity Reference Database (ToxRefDB). The authors profiled results from *in vivo* chronic toxicity/carcinogenicity studies across 310 chemicals currently contained within ToxRefDB. This database has been suggested to be a model for development of predictive signatures of toxicity and for validation of the U.S. EPA’s ToxCast research program (Martin et al. 2009). Although the goals of the U.S. EPA program are worthy of our support because they promote both the prediction of response for chemicals with unknown activity and the avoidance of unnecessary testing in animals, CropLife America has several concerns regarding the application and use of ToxRefDB, as described by Martin et al.

First, we at CropLife America understand that ToxRefDB is not intended for risk assessment purposes. However, in their Table 3, Martin et al. (2009) present a list of 109 chemical compounds according to a “relative potency” grading system (using a scale based on lowest-effect dose levels/end points from chronic toxicity studies) and whether or not tumors occurred according to multigenders/multisites/multispecies. Thus, the summary presented in Table 3 represents a hazard ranking system based on relative potency. Such systems are used as tools by some regulatory bodies to make decisions (e.g., the European Union, the State of California under Proposition 65). As a result, it is possible, and even likely, that the data in Table 3 could be used to support regulatory action based solely on this relative potency ranking, which is outside the context of the formal risk assessment process. Even a cursory review of the chemicals listed in Table 3 reveals the presence of many currently registered food-use pesticides that have not been found to pose any unacceptable cancer risk. Therefore, additional care should be taken in the future with regard to any potential rankings using ToxRefDB analyses.

Second, it is problematic that some data entries for chemicals listed in Table 3 (Martin et al. 2009) demonstrate an absence of available information. For example, we checked the accuracy of the Table 3 entry of “N” indicating “not assessed (no study available).” This entry appeared for 12 chemicals (pesticides) that denoted a lack of multi species carcinogenicity data. In six cases (i.e., pyraclostrobin, dichlorvos, alachlor, captan, maneb, and propargite) the U.S. EPA website of Reregistration Eligibility Decision (RED) documents (U.S. EPA 2009) indicates that, contrary to information presented in Martin et al.’s Table 3, multispecies carcinogenicity studies are available. This inconsistency involving readily available information from RED documents and from data evaluation records (DERs; used primarily by the ToxRefDB) should be addressed to ensure future consistency and completeness of the data. This additional check of existing information by the ToxRefDB is especially important given the statement by Martin et al. (2009) in their abstract that “these data are now accessible and mineable within ToxRefDB and are serving as a primary source of validation for U.S. EPA’s ToxCast research program in predictive toxicology.” Any validation work performed with an incomplete database would be questionable.

Third, we would prefer greater transparency when analyzing “cancer datasets” involving grouping of nonneoplastic proliferative lesions with preneoplastic lesions/neoplastic lesions. Because this type of grouping of non-neoplastic proliferative lesions with neoplasia is not standard evaluation practice (Williams et al. 2008), we at Crop Life America would prefer future ToxRefDB interpretation to identify specific terminology used with regard to scoring system(s) of end point progression schema [such as used by Martin et al. (2009) in their Figure 3A]. Identification of specific terminology and key events for non neoplastic proliferative lesions and pre-neoplastic lesions would increase transparency for future publications. Martin et al. stated in their “Results” that they used their method to increase species concordance, which in turn, probably increased the relative power of the statistical analysis. However, it is well known that hyperplastic lesions, for instance, do not always progress to tumors (Klaunig and Kamendulis 2007). By grouping nonneoplastic proliferative lesions with neoplasia, Martin et al. may have increased species concordance and statistical power but may have failed to fully consider the biological plausibility and/or consequences of the grouping. Moreover, the use of this controversial application in the interpretation of the analyses presented by Martin et al. casts some doubt on their validity.

Finally, our last concern involves implementation of results from *in vivo* testing, which Martin et al. (2009) compared with results of *in vitro* testing. The authors failed to discuss the issue of chemical activation and detoxification. It is a general principle of toxicology that toxicity of chemicals can be directly dependent on metabolism (Kemper et al. 2008). Metabolic pathways exist in intact animals but not in isolated cells. Therefore, both pharmacokinetics and metabolism are important biological components of the toxicity profile of any chemical. It is not clear that the current analyses using the ToxRefDB (Martin et al. 2009) took this into consideration.

In conclusion, based on our four concerns given above, we hope that the authors will address these points to increase the degree of clarity and consistency of interpretation of analyses using the ToxRefDB.
